# Rethinking psychometrics through LLMs: how item semantics shape measurement and prediction in psychological questionnaires

**DOI:** 10.1038/s41598-025-21289-8

**Published:** 2025-10-24

**Authors:** Federico Ravenda, Antonio Preti, Michele Poletti, Antonietta Mira, Andrea Raballo

**Affiliations:** 1 Euler Institute, Faculty of Informatics, Università della Svizzera italiana, Lugano, Switzerland; 2 Department of Neuroscience, University of Turin, Turin, Italy; 3 Department of Mental Health and Pathological Addiction, Child and Adolescent Neuropsychiatry Service, Azienda USL-IRCCS di Reggio Emilia, Reggio Emilia, Italy; 4 Euler Instituite, Faculty of Economics, Università della Svizzera italiana, Lugano, Switzerland; 5 Department of Science and High Technology, Insubria University, Como, Italy; 6 Chair of Psychiatry, Faculty of Biomedical Sciences, Università della Svizzera italiana, Lugano, Switzerland; 7 Cantonal Sociopsychiatric Organisation, Public Health Division, Department of Health and Social Care, Repubblica e Cantone Ticino, Lugano, Switzerland; 8 Euler Institute, Faculty of Biomedical Sciences, University of Lugano, Lugano, Switzerland

**Keywords:** Psychological Questionnaire, Large Language Models, Psychometrics, Psychology, Computational science, Psychiatric disorders

## Abstract

Psychological questionnaires are typically designed to measure latent constructs by asking respondents a series of semantically related questions. But what if these semantic relationships, rather than reflecting only the underlying construct, also impose their own structure on the data we collect? In other words, to what extent is what we “measure” in questionnaires shaped a priori by item semantics rather than revealed solely a posteriori through empirical correlations? To examine this epistemological question, we propose LLMs Psychometrics, a novel paradigm that harness LLMs to investigate how the semantic structure of questionnaire items influences psychometric outcomes. We hypothesize that the correlations among items partly mirror their linguistic similarity, such that LLMs can predict these correlations-even in the absence of empirical data. To test this, we compared actual correlation matrices from established instruments—the Big 5 Personality (Big 5) and Depression Anxiety Stress Scale (DASS-42)—with the semantic similarity structures computed by LLMs. Among the top 3 semantically similar items, the empirically most correlated item was found in 95% of DASS cases and 82% of Big 5 cases. Building on this, we developed PsychoLLM, a neural proof-of-concept architecture, which uses item semantics to predict responses to new items–demonstrated with the Generalized Anxiety Disorder-7 (GAD-7) and Patient Health Questionnaire-9 (PHQ-9). PsychoLLM achieved  70% accuracy when predicting one scale’s responses from the other, enabling new analyses based on semantic relationships. This work underscores an important epistemological implication for psychometrics: item semantics may influence measurement outcomes to varying degrees, more extensively than previously assumed. By leveraging LLMs to expose this a priori semantic structure, researchers can refine questionnaire design, assess data quality, and expand interpretive possibilities, ultimately inviting a reexamination of “*what*” and “*how*” we truly measure in psychology.

## Introduction

In psychology and mental health sciences, measurements are essential for testing theories and evaluating hypotheses. However, whereas in the hard sciences (e.g., physics, chemistry, biology), measurement typically involves fixed units and ratio scales–such as measuring voltage in volts or mass in kilograms–in the soft sciences, such as psychology and the broader social and behavioral sciences, measurement often relies on ranking responses or using scaling techniques. This is because psychological constructs like anxiety, intelligence, or depression are inherently abstract, latent, and cannot be observed directly. Unlike physical phenomena, which can be measured independently of their definitions, psychological constructs are deeply tied to how they are theoretically defined and operationalized. This introduces unique challenges, such as ensuring that measurement tools align with the intended construct and addressing potential biases introduced by cultural, contextual, or semantic factors.

Measurement in psychology generally involves the systematic assignment of numerical values to objects or events according to specific rules^1^. Although widely accepted and taken for granted, this fundamental assumption, i.e. that psychological measurement captures objective properties of mental constructs, needs critical examination. In psychology, this often results in the use of scales, such as Likert’s, that assess the intensity of an individual’s feelings or attitudes through responses to structured questionnaires. However, these tools depend heavily on participants’ subjective interpretations. Individual responses can therefore be greatly influenced^2^ either by personal biases^3–5^ or simple misunderstandings of the questions. This subjectivity may obscure the true nature of the constructs being measured, as responses may reflect more the perceptions of participants or the influence of the testing environment than the constructs themselves.

The changing and context-sensitive nature of psychological states and traits adds extra complexity. Traits are not static; they can change over time and be affected by different external factors^6,7^. This variation makes it challenging to keep measurements of constructs consistent over time or across different populations. More fundamentally, the very act of measurement through language may impose an a priori structure on what we claim to measure empirically.

Psychological tests typically consist of items or questions that are designed to assess specific aspects or properties of the measured construct, including attitudes^8^, traits^9^, and emotions^10^. These items are carefully crafted to capture different facets or dimensions of the construct^11^, based on theoretical definitions or empirical evidence. Each item in a psychological test serves as a behavioral indicator or expression of the underlying construct. Responses to items may be fundamentally structured by their semantic relationships, questioning our assumption that we are primarily measuring latent psychological traits.^12^. Therefore, the items indirectly provide a definition or operationalization of the construct, as they reflect observable manifestations or characteristics associated with it^13^. Overall, the items in a psychological test play a crucial role in defining and operationalizing the construct, as they serve as the primary means of measurement and assessment.

Additionally, since many psychological traits are latent, they must be measured indirectly through proxies such as responses to survey questions or observed behaviors. These indirect measurements require sophisticated techniques such as factor analysis^14,15^ or item response theory^16^ to analyze the patterns of responses on Likert scales and derive meaningful information about the underlying psychological constructs being measured. These methods help researchers understand the structure of the construct and interpret the responses, despite the lack of a direct ratio measurement.

The evolution of psychometric methods has taken a significant turn with the use of natural language processing (NLP) techniques^17–23^. Specifically, Large Language Models (LLMs) can be used to extract semantic embeddings—dense vectors that capture and synthesize the semantic content of textual data—to represent questionnaire items. These embeddings allow us to quantify the semantic relationships between items by encoding their meaning in a high-dimensional space, providing a mathematical foundation for analyzing the linguistic structure of psychological measures.

Indeed, according to the unfolding theory^24^, individuals’ responses to items are influenced not only by their true level on the latent trait and the characteristics of the items (such as difficulty) but also by the semantic content of the items. The wording, clarity, and relevance of items can affect how people understand and respond to them. Clear and well-aligned survey items can elicit more accurate and consistent responses from individuals. Conversely, ambiguous or poorly constructed items may result in more variable and unreliable responses. This raises a crucial epistemological question: to what extent do psychological questionnaire create the very constructs they claim to measure?

By integrating LLMs into psychometric evaluations, researchers can achieve a more accurate and contextually relevant assessment of psychological traits. We will call this emerging field, which leverages the capabilities of LLMs to enhance psychometric methodologies, as “*Large Language Model Psychometrics*”.

**Fig. 1:**
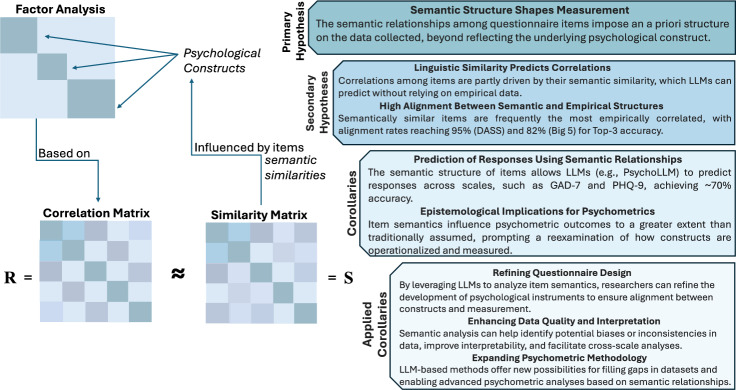
Overview that presents the conceptual framework of the logical progression from the overarching hypothesis, linking traditional factor analysis with semantic similarity analysis in psychometric research, illustrating how LLMs can enhance measurement through the relationship between empirical correlations (R) and semantic similarity matrices (S).

We refer to Fig. 1 for a visual synthesis of the logical progression from the overarching hypothesis to specific implications and practical applications, highlighting the transformative potential of integrating LLMs in psychometric research. For a schematic overview of the challenges faced in psychological testing and core psychometric assumptions, see Section A in Supplementary Material.

**Fig. 2:**
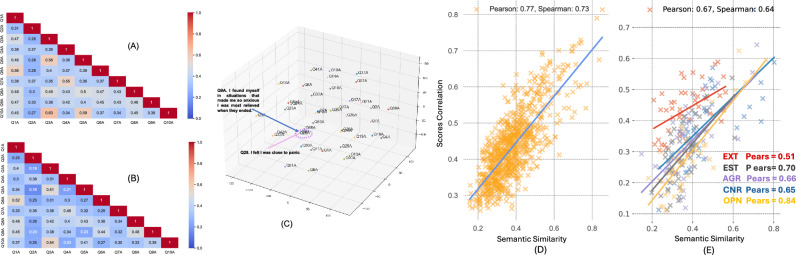
All the plots refer to the results obtained using GPT$_{large}$ as semantic model. **(A)** represents the correlation matrix of the scores associated with the first 10 items of the DASS scale, while **(B)** shows the corresponding semantic similarities for these items. **(C)** represents embeddings related to the items of the DASS scale in 3 dimensions, after applying a dimensionality reduction algorithm (t-SNE) for visualization purposes. **(D)** and **(E)** are scatter plots where the x-axis represents the semantic similarity between items, and the y-axis represents the corresponding correlation between scores. In (E), the correlation is calculated conditioned on the five different factors of the Big 5 scale and then averaged.

## Methods and results

### Datasets

The data used in this work are sourced from the **Openpsychometrics** repository, accessible via the following link: https://openpsychometrics.org/_rawdata/. This archive offers a collection of interactive personality tests, where individuals can complete self-reported questionnaires.

For this analysis, two questionnaires related to the Big 5 Personality^25^ (1’015’341 respondents) and the Depression Anxiety Stress (DASS) (39’775 respondents) scales^26^ have been used. Data are presented in a tabular format: each row corresponds to the responses of an individual, and each column represents an item of the questionnaire, in addition to some demographic information of the individual, which has been excluded from the considered work. The choice to use a publicly available dataset was made to ensure maximum reproducibility and transparency.

Additionally, for a specific task where we predict responses to one questionnaire given the responses to another, we used data from Wave 6 of the COVID-19 Psychological Research Consortium (C19PRC) study^27^. This data come from a longitudinal online survey, initiated in March 2020, that monitors the psychological, social, and economic impact of the COVID-19 pandemic on the UK adult population. The study employs validated self-report questionnaires to assess various aspects of participants’ experiences. Participants provided information about their mental health conditions (depression, anxiety, and COVID-19 related PTSD), physical health status, psychological characteristics, social and political attitudes, COVID-19 related experiences, and demographic information. Specifically, we focused on participants’ responses to two validated mental health questionnaires: the Generalized Anxiety Disorder-7 (GAD-7) and the Patient Health Questionnaire-9 (PHQ-9). The data is publicly available through the Open Science Framework (OSF) https://osf.io/v2zur/.

For reproducibility purpose, we have made our analysis code freely accessible. All materials, including the raw data and the complete codebase used to perform our analyses, are available in a public GitHub repository: https://github.com/Fede-stack/Exploiting-semantic-structure-in-psychological-questionnaire/tree/main.

Two types of information are considered: the first has semantic nature—each item represents textual information that can be used to assess the similarity between different items—while the second one has numerical nature—each item for each individual is marked with a numerical score that reflects the intensity of the response to the question.

### Problem formulation

In this work, we explore how data from questionnaires can be used together with LLMs and how to benefit from this approach to enhance psychometric evaluations. Data are represented in a tabular format, where each column corresponds to an item, each row to an individual, and each cell contains the individual’s response to a specific item. Typically, approaches such as factor analysis are used to identify factors within the scale based on the correlation scores among the items’ scores. However, this study extends beyond conventional methods by demonstrating that the semantic structure of the items can account for a significant portion of the variability in the data, which means that the similarity structure reflects the correlation structure:

$$\begin{aligned} sim(item_i, item_j) \approx \rho (S_i, S_j) \end{aligned}$$

where $sim(item_i, item_j)$ represents the semantic similarity between items $i$ and $j$, and $\rho (S_i, S_j)$ denotes the correlation between their respective scores.

Building on this foundation, we integrate semantic information (the textual content of items) with numerical data (the scores), adapting LLMs for score prediction. This approach, called PsychoLLM, allows for enhanced score predictions accuracy:

$$\begin{aligned} PsychoLLM({\textit{items}}, {\textit{scores}}) \rightarrow Y \end{aligned}$$

Here, $PsychoLLM(\text {items}, \text {Scores})$ model is a function that maps items and their associated scores into a value $Y$, the outcome variable, which could be the score of a missing imputation or a new item.

### Step 1: Items’ semantic structure as a proxy for scores’ structure

**Table 1:** The columns represent the Top-1, Top-2, and Top-3 accuracy metrics. The Pearson (*r*) and Spearman ($\rho$) coefficients are reported for both questionnaires. “$^*$” denotes the significance of the coefficients (p-value $< 0.05$). In all the cases the higher the score, the better (highlighted in bold).

Models	Top-1 (%)	Top-2 (%)	Top-3 (%)	$\textbf{r}$	$\mathbf {\rho }$
DASS (42 items)					
all-MiniLM-L6-v2	45.2%	61.9%	69.0%	$0.63^{*}$	$0.62^{*}$
all-MiniLM-L12-v2	50.0%	66.7%	73.8%	$0.63^{*}$	$0.62^{*}$
all-mpnet-base-v2	57.1%	71.4%	80.1%	$0.67^{*}$	$0.66^{*}$
RoBERTa	45.2%	52.4%	59.5%	$0.59^{*}$	$0.59^{*}$
DistilBERT	19.0%	47.6%	54.8%	$0.64^{*}$	$0.65^{*}$
BERT	35.7%	45.2%	54.8%	$0.50^{*}$	$0.57^*$
GPT$_{small}$	61.9%	90.5%	92.9%	$0.74^{*}$	$0.70^{*}$
GPT$_{large}$	**71.4%**	**88.1%**	**95.2%**	${\textbf {0.77}}^{*}$	${\textbf {0.73}}^{*}$
GPT$_{ada}$	64.3%	81.0%	85.7%	$0.75^{*}$	$0.73^{*}$
T5	64.3%	85.7%	88.1%	$0.65^{*}$	$0.59^{*}$
Big 5 Personality (50 items)					
all-MiniLM-L6-v2	58.0%	66.0%	68.0%	$0.61^{*}$	$0.57^{*}$
all-MiniLM-L12-v2	56.0%	62.0%	66.0%	$0.59^{*}$	$0.52^{*}$
all-mpnet-base-v2	54.0%	66.0%	72.0%	$0.53^{*}$	$0.43^{*}$
RoBERTa	36.0%	48.0%	54.0%	$0.50^{*}$	$0.46^{*}$
DistilBERT	36.0%	38.0%	46.0%	$0.42^{*}$	$0.42^{*}$
BERT	8.0%	12.0%	14.0%	$0.20^{*}$	$0.18^*$
GPT$_{large}$	58.0%	72.0%	**82.0%**	$0.67^{*}$	${\textbf {0.64}}^{*}$
GPT$_{small}$	54.0%	72.0%	78.0%	${\textbf {0.69}}^{*}$	${\textbf {0.64}}^{*}$
GPT$_{ada}$	54.0%	**74.0%**	76.0%	$0.63^{*}$	$0.57^{*}$
T5	**60.0%**	72.0%	78.0%	$0.64^{*}$	$0.57^{*}$

For each item in the questionnaire, a numerical vector is created, known as a *embedding*, capable of synthesizing the semantic information of each question. To generate these embeddings, we explored different state-of-the-art pre-trained language models (PLMs):

1. *Encoder-based models*:
  BERT family: *BERT*, *RoBERTa*, and *DistilBERT*^28–30^. These models produce contextual word representations, capturing the subtle meanings of words based on their surrounding context.
  Sentence-BERT (SBERT) models: *all-MiniLM-L6-v2*, *all-MiniLM-L12-v2*, and *all-mpnet-base-v2*^31^. These models are specifically designed to generate sentence-level representations, optimized for semantic similarity tasks and efficient retrieval operations.
2. *OpenAI’s embedding models*: *text-embedding-3-small*, *text-embedding-3-large*, and *text-embedding-ada-002*, hereafter referred to, respectively, as GPT$_{small}$, GPT$_{large}$, GPT$_{ada}$. These models are Transformer-based architectures, derived from the GPT (Generative Pre-trained Transformer) family^32^. They are designed to create high-quality semantic embeddings.
3. *Encoder-decoder model*: T5 (Text-to-Text Transformer)^33^. For our embedding generation task, we also use T5 transformer to create semantic representations of the input text. T5 unique architecture allows it to capture both local and global context, potentially offering a different perspective on semantic relationships compared to pure encoder or decoder models.

To calculate the semantic similarity between items (represented as embeddings) we use cosine similarity (where a higher cosine similarity corresponds to greater similarity, values can range from -1 to 1). The cosine similarity is calculated as follows:

$$\text {cosine similarity}(\text {item}_i, \text {item}_j) = \frac{\text {item}_i \cdot \text {item}_j}{\Vert \text {item}_i\Vert \Vert \text {item}_j\Vert }$$

where, $\text {item}_i \cdot \text {item}_j$ represents the dot product of the two item’s vectors, and $\Vert \text {item}_i\Vert$ and $\Vert \text {item}_j\Vert$ are the Euclidean norms of the vectors $\text {item}_i$ and $\text {item}_j$, respectively.

The assumption we make is that there is a link between semantically similar items and the correlation of their scores. Building upon this, the use of PLMs facilitates a sophisticated and detailed comprehension of textual data in questionnaires.

In Fig. 2C, an example of the close distribution of item embeddings in a 3-dimensional space when examining the DASS scale can be seen: representations of items **Q28.** “*I felt I was close to panic*” and **Q9.** “*I found myself in situations that made me so anxious I was most relieved when they ended*” are very close in the embedding space (after applying the t-SNE algorithm^34^ for dimensionality reduction). In Supplementary Table 3, for each item of the DASS questionnaire, the 3 most semantically similar items, according to GPT$_{large}$ embedding model, are shown. This table reveals many significant semantic redundancies among the considered items, particularly between those identified as most similar. These insights have important implications for questionnaire design: the observed semantic overlap suggests that certain items may be capturing similar aspects of mental states, potentially leading to redundancy in the assessment.

Table 1 shows results based on different PLMs. The overall most effective embedding models across both datasets are GPT$_{large}$ and GPT$_{small}$.

In evaluating the relationship between semantic similarity and item correlation, we employ the concept of Top-K accuracy. This metric measures the frequency with which the most correlated item appears within the top *K* most semantically similar items, thus providing a quantitative measure of how well semantic similarity aligns with item correlations across the psychological questionnaire.

In the DASS scale analysis, 30 out of 42 items show that the highest correlation (every correlation value is considered in absolute terms) corresponds with the greatest semantic similarity between the items. For the Big 5 Personality scale, this pattern is observed for 30 out of 50 items. The *Top-1 Accuracy*, *Top-2 Accuracy*, and *Top-3 Accuracy* columns in Table 1 shows the cumulative percentage of items where the highest correlated item appears within, respectively, the top one, top two, and top three positions. For the GPT$_{large}$ model, we observe that in 95.2% of cases, the model correctly matched items with the highest score correlations to the top three semantically similar items on the DASS scale. On the Big 5 Personality scale, this accuracy is about 82.0%.

**Fig. 3:**
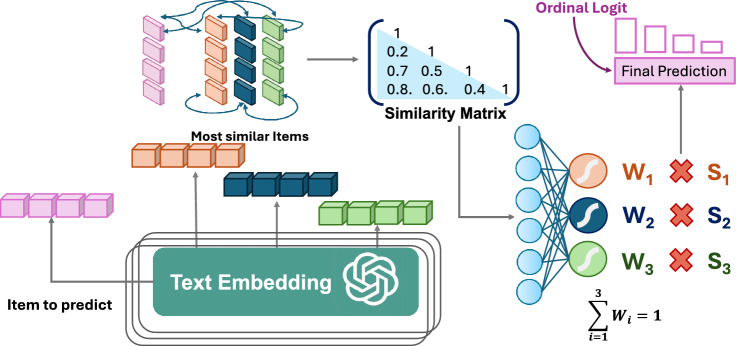
Overview of the main steps of the PsychoLLM architecture.

Furthermore, the Pearson (*r*) and Spearman ($\rho$) correlation between the correlation matrix and the semantic similarity matrix are calculated and statistically tested (using a t-test, $\alpha = 0.05$). The two coefficients differ in their approaches: Pearson correlation measures the linear relationship between two variables assuming normal distribution, while Spearman correlation assesses the monotonic relationship, making it suitable for non-parametric data where the relationship is not necessarily linear. The coefficients are positive for both scales (particularly high for the DASS scale). The Pearson coefficient for the DASS scale, using GPT$_{large}$ model, is 0.77, while the Spearman coefficient is 0.66 (see Fig. 2D for a visual representation). For the Big 5, we average the coefficients computed separately for the five factors: the best model are GPT$_{large}$ and GPT$_{small}$ where the Pearson coefficient is respectively 0.67 and 0.69, whereas the Spearman coefficient is 0.64 in both cases (see Fig. 2E for a visual representation). In all the cases, the two coefficients indicate a positive correlation: *the semantic similarity matrix between items seems to reflect well the correlation of the scores*, especially for the DASS scale As an additional proof-of-concept, for the DASS scale, we show in Supplementary Table 1 how the factor analysis based on the semantic matrix reflects the same factor structure as the one calculated based on the correlation matrix. These results demonstrate that there is positive and statistically significant correlation between the scores associated with the items and the semantic similarity of the items themselves. Additionally, Fig. 2 presents the correlation (A) and semantic similarity (B) matrices for the first 10 items of the DASS scale, showing how the two heatmap-structures appear to be very similar to each other.

These findings raise a fundamental question about the nature of factor analysis in psychological measurement. When semantic similarity matrices demonstrate such high correlations with empirical data, and LLMs can effectively predict item correlations without any empirical observations, we face a novel interpretation: rather than solely uncovering latent psychological constructs, factor analysis might be substantially capturing the semantic relationships inherently embedded in the measurement instruments themselves. Such an interpretation challenges traditional assumptions about psychological measurement and suggests that the structure we discover through factor analysis may be, at least in part, a reflection of the linguistic framework we use to construct our assessment tools. In Section B in Supplementary we prove how factor analysis based on semantic similarity matrix aligns with traditional factor analysis results for DASS-42 scale.

### Exploiting psychological questionnaire through PsychoLLM

Our previous empirical findings demonstrate that the semantic structure significantly correlates with the score structure within psychological questionnaires. This observation allows us to reason that, once the semantic structure has been explored, it becomes easier to exploit the score structure of a psychological questionnaire.

Building on these insights, we developed PsychoLLM, a lightweight neural architecture that combines semantic analysis with questionnaire scoring methodology. Our model leverages both semantic similarities between questionnaire items and actual questionnaire scores, enabling two key capabilities: (1) predicting scores for new items based on their semantic content, and (2) predicting responses to one questionnaire given the responses to another.

**Fig. 4:**
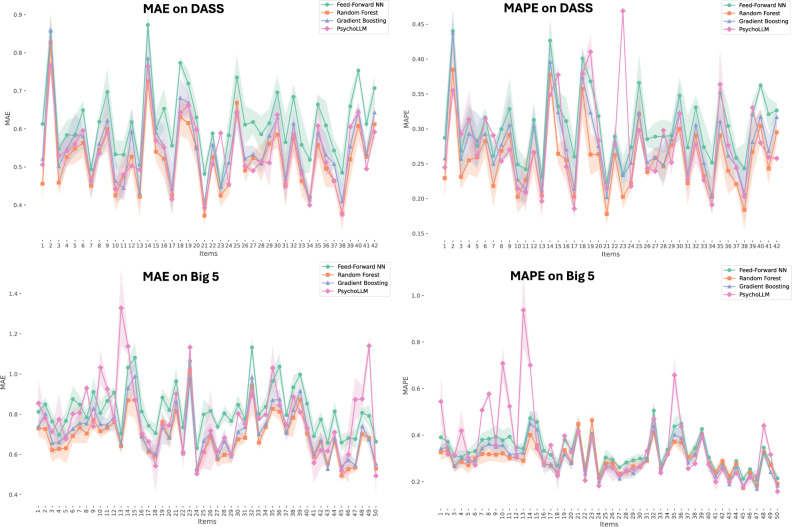
MAE and MAPE scores of PsychoLLM and the considered supervised methods are shown for the items of the questionnaires considered, Big 5 Personality and DASS. The shaded bands around each line represent the standard deviation of the metric values for each questionnaire item.

The core concept of our architecture, shown in Fig. 3, is to establish relationships between similarity structures in the following way: for each item in the questionnaire, we identify the three most semantically similar items. We choose three items because, as shown in Table 1, this number typically encompasses the item with the highest correlation for the vast majority of cases. Using these semantically similar items, we construct a similarity matrix based on GPT$_{large}$ embeddings, which achieved the best performance in Table 1. The similarity matrix is fed into two fully connected layers, which estimate weights used to adjust the scores of the most similar items. This method allows us to exploit semantic relationships to inform score predictions, potentially capturing subtleties that traditional scoring methods may overlook. A detailed explanation of the implementation, including the specifics of the neural network architecture and training process, can be found in Section D and Supplementary Table 2.

In the following subsection, we demonstrate how the semantic theory of questionnaires can be used to predict scores for new items.

### Step 2: predicting a new item scores

For this specific task, we used data from the Big 5 Personality and Depression Anxiety Stress Scales questionnaires. Given that the number of observations in each dataset is exceptionally large compared to the typical sample sizes observed in standard studies^35^, we randomly sampled 1,000 observations from each dataset for our analysis.

Considering a scenario where the aim is to predict a patient response to a new question based on their answers to other items: in traditional settings, when a model is trained on a subset of variables, it is unable to predict a score for an item on which it has not been trained. A simplistic method might involve using a summary measure (e.g., mean, median, or mode) derived from responses to other questions. However, our approach goes beyond this by establishing connections between the scores of different items and their semantic similarities.

The relationship between the correlation of scores and the semantic similarity between items in a questionnaire has been empirically demonstrated in Section 2.3. To demonstrate we can predict in a unsupervised way the score of a new question, an item is removed from the original dataset, its score is predicted, and then compared with the actual observed score for validation. The idea of these models is to create associations between semantic similarities and scores of similar semantic entities.

In Table 2, the results obtained from PsychoLLM are summarized together with supervised approaches (i.e., where the true observed value for the specific item to be predicted is used to guide learning during the training phase). The final result represents the average of the final items’ scores. As benchmarks, we consider a Feed-Forward Neural Network (NN), and two ensemble models, Random Forest^36^ (RF), and Gradient Boosting^37^ (GB) (results are averaged after a 5-fold cross-validation is performed). For each iteration, two metrics are calculated to evaluate the goodness of the model: the Mean Absolute Error (MAE), which measures the average absolute difference between predictions and actual values, and Mean Absolute Percentage Error (MAPE), which expresses this error as a percentage, offering both an intuitive error measure (MAE) and a relative scale-dependent one (MAPE) (see Section C for a discussion on the metrics used). Subsequently, the scores are averaged and compared with those obtained from the self-supervised PsychoLLM model.

For the DASS questionnaire, PsychoLLM achieves a Mean Absolute Error (MAE) of 0.54 and a Mean Absolute Percentage Error (MAPE) of 0.28, which is comparable to the best performing supervised methods. MAE of 0.54 is particularly low given that the DASS questionnaire uses a 4-point Likert scale. In this context, an average error of just over half a point demonstrates strong predictive power, as it indicates that the model’s predictions are, on average, within one scale point of the actual responses. For the Big 5 Personality questionnaire, PsychoLLM shows a MAE of 0.78 and MAPE of 0.35, slightly higher than the supervised ensemble methods. It is important to highlight that PsychoLLM achieves these results without the need for labeled training data, unlike the supervised methods (Feed Forward NN, Gradient Boosting, and Random Forest). Supervised approaches are implemented using their default hyperparameters as provided in the scikit-learn library to ensure reproducibility.

**Table 2:** Comparison of MAE and MAPE values for *Predicting New Items* task. For PsychoLLM, GPT$_{large}$ is used to generate the embeddings. Scores are averaged across all the new-item predictions.

Models	DASS		Big 5 Personality	
	**MAE**	**MAPE**	**MAE**	**MAPE**
**Predicting New Items**				
PsychoLLM	0.54	0.28	0.78	0.35
Feed Forward NN	0.62	0.30	0.82	0.33
Gradient Boosting	0.55	0.28	0.72	0.30
Random Forest	0.53	0.26	0.70	0.30

This process is repeated for each item in the questionnaire, and the final results reported in Table 2 represent the average of the metrics calculated for each item. We call this approach Leave-One-Item-Out (LOIO) method. For this reason, we do not test any hypotheses between the models presented in Table 2, as the results refer to predictions made for different items. Regarding the DASS dataset, the PsychoLLM model often outperforms supervised models. This result is particularly surprising, as it frequently surpasses benchmarks without ever seeing the actual response values, but instead infers them solely based on the similarity matrix and the scores associated with the most similar items. Moreover, considering the prediction on individual items for the DASS scale, PsychoLLM performs better than the corresponding supervised NN, which only considers the scores as input, in *41 out of 42 cases* in terms of **MAE** and in *32 out of 42 cases* in terms of **MAPE**, while it shows similar results to RF and GB. In Fig. 4 we can observe the scores w.r.t. MAE and MAPE for each item of the two considered questionnaire. For the Big 5 dataset, the difference between PsychoLLM’s scores and those of the two ensemble methods appears to be slightly worse overall than for the DASS dataset. However, as seen in Fig. 4, PsychoLLM proves to be highly competitive across many items, often outperforming the supervised methods. Nevertheless, for certain items, the model struggles to accurately predict scores.

These results can be viewed from two different perspectives, each of which provides important insights into the nature of psychological questionnaires.

Firstly, these findings serve as a proof of concept for our earlier assertion that the semantic component alone is sufficient to explain most of the variability in questionnaire data. The competitive performance of PsychoLLM, an unsupervised method relying primarily on semantic information, demonstrates that the linguistic structure of questionnaire items captures much of the underlying psychological constructs being measured. This reinforces our understanding of how language and meaning play a crucial role in shaping respondents’ answers, going beyond the content of the questions themselves.

Second, these results indicate the development of a useful tool for the administration and analysis of questionnaires in the real world. In contexts where traditional data collection methods may be limited, time-consuming or expensive, PsychoLLM offers several practical applications. Researchers can use this approach to estimate how respondents might answer new, semantically related questions without the need for additional data collection, which could be particularly useful in questionnaire development and refinement processes.

Finally, to address potential concerns about item redundancy within subscales making prediction overly straightforward, we conducted additional validation using a more stringent Leave-One-Subscale-Out (LOSO) approach on the DASS-42 scale. In this evaluation, we systematically removed all items from one subscale (Depression, Anxiety, or Stress) and attempted to predict their scores using only items from the remaining two subscales. We focused this analysis on DASS-42 due to its well-established three-factor structure with clearly defined and empirically validated subscales that are theoretically related yet sufficiently distinct to provide a meaningful test of cross-construct prediction.

**Fig. 5:**
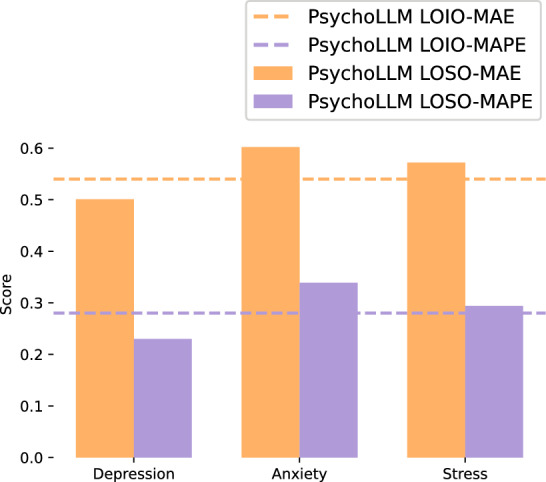
Comparison of Leave-One-Item-Out (LOIO) and Leave-One-Subscale-Out (LOSO)—conditioned on Depression, Anxiety, and Stress subscales—performance on DASS-42.

The LOSO results in Fig. 5 demonstrate that PsychoLLM maintains robust performance even under these challenging conditions. For Depression items predicted from Anxiety and Stress subscales, we achieved MAE of 0.50 and MAPE of 0.23. Anxiety items showed MAE of 0.60 and MAPE of 0.34, while Stress items yielded MAE of 0.57 and MAPE of 0.34. These results are remarkably similar to our LOIO performance, indicating that PsychoLLM’s predictive capability extends beyond within-subscale redundancy to capture meaningful semantic relationships across different psychological constructs. Interestingly, we observed differential predictability across subscales, with Depression items being somewhat easier to predict from other constructs, likely reflecting the stronger semantic overlap between depression and anxiety/stress symptoms in clinical literature.

### Step 3: cross-questionnaire response prediction using semantic similarity

As a further proof-of-concept of our study, we investigated whether it’s possible to predict responses to a new psychological questionnaire based solely on responses to a related one. For this purpose, we used responses to the GAD-7 and PHQ-9 questionnaires. Both questionnaires are used to assess similar constructs related to anxiety and depression, respectively.

In this scenario, we employed the same approach implemented in the previous subsection: We trained PsychoLLM to predict scores for semantically similar items, then made predictions for new items based only on their semantic similarity to the items used in training (considering the three most similar training items for each test item). In this case, we used one questionnaire for training and the other for testing, and vice versa.

Since questions in both questionnaires are impersonal (e.g., GAD2 “*Not being able to stop or control worrying*”, PHQ4 “*Feeling tired or having little energy*”), we made a small modification to convert them into first-person statements to better align with our experimental setup (e.g., GAD2 “*I am not able to stop or control worrying*”, PHQ4 “*I feel tired or have little energy*”). For reproducibility, the complete list of items has been added to the Supplementary Table 4.

The results are shown in Table 3. We observed that the choice of which questionnaire to use for training versus testing does not significantly impact performance, with results being very similar in both scenarios.

When using PHQ-9 for training and GAD-7 for testing, we observed a MAE of 0.34 and a MAPE of 0.16, extremely low values on a 4 Likert scale. Furthermore, in 71% of cases, the score predicted by PsychoLLM exactly matched the observed scores.

In the opposite scenario—using GAD-7 for training and PHQ-9 for testing—we observed a MAE of 0.36 and a MAPE of 0.20. In this case, 70% of predicted scores exactly matched the observed values.

Beyond mere correlation, this proof-of-concept empirically demonstrates how leveraging semantic similarity structure can yield practical predictive power, where responses to one questionnaire can be accurately predicted from another based solely on the semantic relationships between their questions.

**Table 3:** MAE and MAPE values for predicting new questionnaires using PsychoLLM (GPT$_{large}$ is used to generate the embeddings). Scores are averaged across 5 runs.

Training Questionnaire	MAE	MAPE
PHQ-9	0.34	0.16
GAD-7	0.36	0.20

## Discussion

The integration of semantic analysis and numerical data in psychometric evaluations has yielded promising results, emphasizing the capability of LLMs to improve traditional assessment methods. Our findings from analyses and proof-of-concept tasks indicate that the semantic structure of questionnaire items strongly correlates with numerical response patterns. This suggests that understanding the textual content of items can offer important perspectives on the underlying response patterns, which traditional psychometric methods might not entirely reflect.

The use of sentence embeddings to represent the semantic information of questionnaire items proved effective in identifying relationships between items and predicting new items scores. The strong relationship between items’ semantic and response patterns, as evidenced by high Top-K Accuracy scores, Pearson and Spearman coefficients, highlights the robustness of this approach. These results reveal a notable pattern: items that are semantically closer, as determined by the language models, tend to have more closely correlated scores. This suggests that respondents who have similar reactions to one item are likely to respond similarly to another item that is semantically related. This observation has significant implications for the design and interpretation of psychological questionnaires. It could lead to more precise and reliable assessments of psychological traits or states. Moreover, this methodology could aid in minimizing redundancy within questionnaires, ensuring each item contributes distinct and meaningful insights to the overall evaluation.

The results obtained from PsychoLLM, a novel and intuitive proof-of-concept model tailored for psychological questionnaires, clearly show how the semantic component is strongly linked to the numerical component within a questionnaire, especially regarding the prediction of new items. Here we model the response to a new item *j* as a combination of the scores and semantics of the most similar items. In Table 2, we show how PsychoLLM is able to correctly predict scores for new items without ever having been trained on them, remaining competitive with, and often outperforming, supervised approaches specifically trained on all the items. It is also observed that the more the variability of the data within a questionnaire is explained by the semantic component (in Table 1, DASS has higher metrics compared to Big 5), the higher the performance on the task of predicting new items.

One of the advantages of this approach is that it can be easily extended to new questionnaires. Future research could further explore the generalizability of this approach across different types of psychological questionnaires. Additionally, by training the model on a large and diverse set of questionnaires, we can enhance its generalization capabilities. This extensive training enables the model to establish numerous connections between items and scores across different contexts, improving its ability to predict responses for entirely new questionnaires.

While this study serves as a proof-of-concept for semantic-based prediction across questionnaires, a limitation concerns the uniform scoring scales used in our analyses. In the cross-questionnaire prediction tasks (GAD-7 and PHQ-9), both instruments employed identical 4-point Likert scales, allowing PsychoLLM to calculate predictions as weighted sums of semantically similar item scores. Future research should address how semantic similarity-based prediction methods can be adapted to handle heterogeneous scoring systems, potentially through scale transformation techniques or normalized scoring approaches. The current findings should therefore be interpreted as preliminary evidence for semantic-based cross-questionnaire prediction under controlled conditions of scale homogeneity, rather than a general solution for all psychometric prediction scenarios.

Overall, this study demonstrates the substantial potential of the new “*Large Language Model Psychometric*” field to advance psychometric evaluations, paving the way for more accurate and reliable assessments.

## Implications for measurement theory in psychology

Our findings fundamentally challenge the traditional view of psychological measurement as a purely empirical process. The discovery that semantic structure predicts empirical correlations questions a core assumption in psychometrics: that factor analysis and similar methods reveal latent psychological constructs through patterns in empirical data.

Instead, our results suggest that the semantic architecture of questionnaires substantially predetermines response patterns. When LLMs can predict item correlations without any empirical data, we must reconsider what factor analysis actually reveals. Are we discovering latent psychological structures, or are we largely recovering the semantic relationships we built into our measurement instruments?

This raises a deeper epistemological question: To what extent do psychological questionnaires create, rather than discover, the constructs they purport to measure? The high predictive power of semantic similarity suggests that response patterns may be more constrained by linguistic structure than previously acknowledged.

These findings necessitate a new theoretical framework for psychological measurement that explicitly recognizes language’s constitutive role. Rather than treating semantic effects as measurement error to be controlled, we must acknowledge them as fundamental to the measurement process itself. This shifts our understanding of psychological questionnaires from neutral tools that reveal pre-existing psychological structures to linguistic frameworks that actively shape how we conceptualize and measure psychological phenomena.

An alternative interpretation of our findings is that LLMs, trained on vast amounts of human-generated text, may not simply replicate linguistic artifacts, but rather capture distributed representations of shared psychological knowledge embedded in language. From this perspective, the observed alignment between semantic and psychometric might highlights the model’s ability to reflect collective human intuitions about psychological constructs. In this view, semantic similarity may be influenced to real psychological patterns as they are expressed through language, and LLMs may function as valid approximations of large-scale human populations.

These two interpretations are not mutually exclusive. Rather, they open a new conceptual space for understanding psychological measurement as situated at the intersection of linguistic form and latent mental structure. This duality necessitates a revised theoretical framework that explicitly recognizes language’s constitutive and reflective roles in psychometrics. Rather than treating semantic effects as bias or measurement error to be controlled, they should be understood as fundamental components of the measurement process.

This reframing has immediate implications for questionnaire development and validation. Traditional psychometric analysis should be complemented with semantic analysis to understand how linguistic structure influences measurement outcomes. PsychoLLM demonstrates how LLMs can support this new approach, offering tools to assess and potentially improve questionnaire design before data collection begins.

## Conclusions

This study reveals a fundamental epistemological challenge in psychological measurement: the structure of questionnaire responses appears to be substantially predetermined by the semantic relationships between items, rather than emerging purely from the measured psychological constructs. Our results demonstrate that Large Language Models can predict response patterns before any data collection, suggesting that what we interpret as measurements of psychological traits may be significantly shaped by linguistic structures inherent in our measurement tools.

This finding fundamentally challenges the traditional psychometric assumption that questionnaire responses primarily reflect underlying psychological constructs. Instead, our semantic theory of questionnaires, validated through PsychoLLM, reveals that response patterns are substantially constrained by the semantic architecture of the questions themselves. This raises crucial questions about what psychological questionnaires actually measure and how we should interpret their results.

The implications extend beyond methodology into the foundations of psychological measurement: if semantic structure predetermines response patterns, we must reconsider our understanding of psychological measurement as an empirical process. This suggests the need for a new epistemological framework that accounts for how language shapes and constrains our measurement of psychological constructs, potentially transforming our approach to psychological assessment and theory development.

Future works can focus on if these findings generalize across languages and cultural contexts. Our analysis focused on English-language instruments. Different languages may structure semantic relationships differently, potentially affecting the alignment between semantic similarity and empirical correlations. For instance, languages with different grammatical structures, cultural concepts of psychological states, or varying levels of semantic precision for emotional terminology might show different patterns of semantic-empirical correspondence. Additionally, cultural variations in response styles, the conceptualization of psychological constructs, and the social acceptability of certain emotional expressions could influence how semantic structures translate into response patterns. Future research should examine whether the LLMs Psychometrics framework holds across diverse linguistic and cultural contexts, potentially requiring culture-specific adaptations of embedding models or different approaches to semantic similarity computation. This cross-cultural validation would be crucial for establishing the universal applicability of our findings and ensuring that semantic analysis tools are appropriate for global psychological assessment.

## Supplementary Information

Supplementary Information.

## Data Availability

All the data are publicly available and the code can be found at the following Github Repository link: https://github.com/Fede-stack/Exploiting-semantic-structure-in-psychological-questionnaire.
