# Supplementary material for: Rethinking psychometrics through LLMs: how item semantics shape measurement and prediction in psychological questionnaires
Source: Sci Rep. 2025 Oct 24;15:37313. doi: 10.1038/s41598-025-21289-8 (PMC12552711; doi:10.1038/s41598-025-21289-8)
Supplement: Supplementary file 1 — Supplementary Information. [file 41598_2025_21289_MOESM1_ESM.pdf]

Supplementary Material of: Rethinking Psychometrics through LLMs: How Item Semantics Shape Measurement and Prediction in Psychological Questionnaires

Federico Ravenda[1*], Antonio Preti[2], Michele Poletti[3], Antonietta Mira[4, 5], Andrea Raballo[6, 7, 8]

[1*]Department of Informatics, Università della Svizzera italiana, Lugano, Switzerland.
[2]Department of Neuroscience, University of Turin, Turin, Italy.
[3]Department of Mental Health and Pathological Addiction, Child and Adolescent Neuropsychiatry Service, Azienda USL-IRCCS di Reggio Emilia, Reggio Emilia, Italy.
[4]Department of Economics, Università della Svizzera italiana, Lugano, Switzerland.
[5]Department of Science and High Technology, Insubria University, Como, Italy.
[6]Chair of Psychiatry, Faculty of Biomedical Sciences, Università della Svizzera italiana, Lugano, Switzerland.
[7]Cantonal Sociopsychiatric Organisation, Public Health Division, Department of Health and Social Care, Repubblica e Cantone Ticino, Lugano, Switzerland.
[8]Faculty of Biomedical Sciences, University of Lugano, Lugano, Switzerland.

*Corresponding author(s). E-mail(s): federico.ravenda@usi.ch;

A Challenges in Psychological Measurement

The measurement of psychological constructs presents several methodological and epistemological challenges, ranging from the subjectivity of measurements to the complexity of data interpretation. The following points highlight the critical aspects that characterize this field of study.

1. **Subjectivity and Bias**:
   - Measurement in psychology relies on tools like Likert scales, which depend on participants' subjective interpretations.
   - Responses can be influenced by **personal biases**, **misunderstandings**, or the **testing environment**, potentially distorting the true nature of constructs.
2. **Indirect Measurement**:
   - Many psychological constructs are latent and measured indirectly through proxies like survey responses or behaviors.
   - Sophisticated techniques (e.g., **factor analysis**, **item response theory**) are needed to derive meaningful insights from indirect data.
3. **Role of Test Items**:
   - Test items not only measure constructs but also operationalize and define them through their **semantic structure**.
   - Ambiguity or poor item design can lead to unreliable or inconsistent responses.
4. **Language as a Constraint**:
   - The process of measurement through language imposes an **a priori structure**, potentially shaping the constructs it seeks to measure.
   - This raises concerns about whether psychological questionnaires **create constructs** rather than measure pre-existing ones.
5. **Advances in Technology**:
   - Natural Language Processing (NLP) and Large Language Models (LLMs) offer new ways to analyze and understand the **semantic content** of test items.
   - LLMs reveal complex patterns in responses, challenging traditional psychometric assumptions and enhancing construct interpretation.
6. **Epistemological Questions**:
   - Psychological assessments may reflect not only the construct being measured but also the influence of item design and semantics, questioning their validity.

Building upon these results, we have developed a proof-of-concept neural architecture that leverages the semantic structure of psychological questionnaires to address several key challenges in psychometric analysis.

B Factor Analysis of DASS-42 Semantic Similarity Matrix

The semantic similarity matrix S of DASS-42 items is used for factor analysis to examine its underlying structure. Given two items i and j, their semantic similarity s_{ij} was computed using GPT_{large} embeddings and cosine similarity. This matrix underwent eigendecomposition:

$$S = V \Lambda V^T$$

where Λ is the diagonal matrix of eigenvalues and V contains the corresponding eigenvectors. The factor loading matrix F was then obtained as:

$$F = V \Lambda^{1/2}$$

To improve interpretability, a varimax rotation was applied to maximize the variance of squared loadings within each factor:

$$F_{rotated} = FR$$

where R is the rotation matrix that maximizes the variance of squared loadings across the three factors (Depression, Anxiety, and Stress) for all 42 DASS items.

The analysis revealed three distinct factors corresponding to Depression, Anxiety, and Stress subscales. Factor structure obtained in Table 1 closely mirrors the established three-factor solution reported in traditional psychometric validations of DASS-42 [1, 2], suggesting that the semantic structure of items inherently reflects their intended psychological constructs a priori.

Items	Subscales' Loadings Depression	Anxiety	Stress
**Depression**			
I felt downhearted and blue	0.55		
I felt sad and depressed	0.51		
I could see nothing in the future to be hopeful about	0.62		
I felt that I had nothing to look forward to	0.76		
I felt that life was meaningless	0.82		
I felt that life wasn't worthwhile	0.85		
I felt I was pretty worthless	0.75		
I felt I wasn't worth much as a person	0.72		
I felt that I had lost interest in just about everything	0.63		
I was unable to become enthusiastic about anything	0.56		
I couldn't seem to experience any positive feeling at all	0.52		
I couldn't seem to get any enjoyment out of the things I did	0.53		0.42
I just couldn't seem to get going	0.40		0.38
I found it difficult to work up the initiative to do things	0.37		0.43
**Anxiety**			
I was aware of the action of my heart in the absence of physical exertion (e.g, sense of heart rate increase, heart missing a beat)		0.57	
I perspired noticeably (e.g. hands sweaty) in the absence of high temperatures or physical exertion		0.57	
I was aware of dryness of my mouth		0.47	
I experienced breathing difficulty (e.g. excessively rapid breathing, breathlessness in the absence of physical exertion)		0.58	
I had difficulty in swallowing		0.43	
I had a feeling of shakiness (e.g. legs going to give way)		0.74	
I experienced trembling (e.g. in the hands)		0.70	
I was worried about situations in which I might panic and make a fool of myself		0.48	
I found myself in situations which made me so anxious I was most relieved when they ended		0.39	0.45
I feared that I would be 'thrown' by some trivial but unfamiliar task		0.42	
I felt I was close to panic		0.61	
I felt terrified		0.59	
I felt scared without any good reason	0.40	0.51	
I had a feeling of faintness		0.69	
**Stress**			
I found it hard to wind down			0.58
I found it hard to calm down after something upset me			0.72
I found it difficult to relax		0.35	0.59
I felt that I was using a lot of nervous energy		0.52	0.41
I was in a state of nervous tension		0.60	0.38
I found myself getting upset rather easily			0.81
I found myself getting upset by quite trivial things			0.77
I found myself getting agitated			0.78
I tended to over-react to situations			0.54
I found that I was very irritable			0.71
I felt that I was rather touchy		0.42	0.48
I was intolerant of anything that kept me from getting on with what I was doing			0.51
I found myself getting impatient when I was delayed in any way (e.g. lifts, traffic lights, being kept waiting)			0.63
I found it difficult to tolerate interruptions to what I was doing			0.61

Supplementary Table 1: Factor loading pattern matrix for the DASS-42, showing the three-factor structure of Depression, Anxiety, and Stress. Values represent standardized factor loadings after varimax rotation. Items are grouped by their theoretical subscales to highlight the alignment between empirical loadings and theoretical constructs.

C Error Metrics

To evaluate the goodness of our model, two error metrics are used: the Mean Absolute Error (MAE), which quantifies the average absolute difference between predicted and observed values, and the Mean Absolute Percentage Error (MAPE), which expresses the error as a percentage of the actual values. MAE offers an intuitive, straightforward measure of the model's error, while MAPE provides a relative measure of error, making it useful for understanding the error in terms of the scale of the data.

$$\text{MAE} = \frac{1}{n}\sum_{i=1}^{n} |y_i - \hat{y}_i|$$

$$\text{MAPE} = \frac{1}{n}\sum_{i=1}^{n} \left|\frac{y_i - \hat{y}_i}{y_i}\right| \times 100$$

where:

- n is the total number of observations.
- y_i represents the actual values or observed points.
- $\hat{y}_i$ represents the predicted values, output by our model.

D Adapting LLMs to Predict New Item's Scores

To predict the score for a given item x, we first identify the three most semantically similar items, x_1, x_2, x_3, using a semantic similarity function based on embeddings. The similarity between the items is computed using cosine similarity.

Architecture and Prediction Process. Once the three most semantically similar items are identified, we construct a similarity matrix between them:

$$S = \begin{pmatrix} 1 & & & \\ s_{21} & 1 & & \\ s_{31} & s_{32} & 1 & \\ s_{p1} & s_{p2} & s_{p3} & 1 \end{pmatrix}$$

where s_{ij} represents the similarity between items x_i and x_j, and p is the item for which we want to predict the score. This matrix is then flattened into an input vector:

$$\text{input} = [s_{21}, s_{31}, s_{32}, s_{p1}, s_{p2}, s_{p3}]$$

The resulting vector is passed through a 10-dimensional Dense layer to handle non-linearities:

$$h = \sigma(W_{\text{hidden}} \cdot \text{input} + b_{\text{hidden}})$$

where W_{hidden} and b_{hidden} are the weights and biases of the feedforward layer, and σ is the gelu activation function [3]. Next, the vector h is passed to a softmax output layer to compute the weights associated with the scores corresponding to the three most similar items:

PsychoLLM's Hyperparameters	Values
learning_rate	0.01
batch_size	32
epochs	3
activation	gelu

Supplementary Table 2: PsychoLLM's hyperparameter parameters used during the training process

$$w_i = \frac{\exp(s_i)}{\sum_{j=1}^{3} \exp(s_j)}$$

where w_i represents the normalized weight for the score of item x_i.

Finally, the computed weights are used to weight the respective scores y_1, y_2, y_3 of the 3 most similar items:

$$\hat{y} = \sum_{i=1}^{3} w_i \cdot y_i$$

The result $\hat{y}$ is then passed to an ordinal logistic layer to take into account the ordinal nature of the scores. The ordinal logistic layer applies the following function for each threshold θ_j, where j is the class of the output:

$$P(Y \leq j|\hat{y}) = \frac{1}{1 + \exp(\theta_j - \hat{y})}$$

This process allows the model to predict the score of the item by considering both the semantic similarity of the items and the ordinal nature of their scores. In Table 2, PsychoLLM's hyperparameters values are shown for completeness.

Self-Supervised Learning Framework. The parameters of the architecture are learned through a self-supervised approach that establishes the relationship between semantic similarity and response prediction without direct supervision on target items. Model's parameters (W_{hidden}, b_{hidden}, and ordinal logistic weights) are optimized through this iterative process using cross-entropy loss between predicted and actual responses. During training, we systematically iterate through each item in the questionnaire, temporarily treating it as a target for prediction while using all other items to learn the semantic-to-response mapping. For each training iteration, we select one item as the target, identify its three most semantically similar items from the remaining set, and train the model to predict the target's responses using only the semantic relationships and response scores of the similar items. This process creates multiple training instances where the model learns generalizable patterns for transforming semantic similarity structures into accurate response predictions across diverse item combinations within the questionnaire.

E Managing Negation in Semantic Analysis of Big 5 Scale

In the context of the Big 5 personality scale, some items are very similar from a semantic standpoint yet hold negative relationships with each other. For example, consider the items "*I don't talk a lot*" and "*I talk to a lot of different people at parties*". Although the semantic content is quite similar, a person scoring high on one is likely to score low on the other.

In such cases, the similarity calculated between the two embeddings will be positive because the semantic content is similar (rather than negative). To address this issue for Big 5, w.r.t. the proof-of-concept *"predicting the new item"* task, we adjusted the way we interpret and process these items in our analysis. The method we used is to reverse-score for all the negatively worded items so that their numerical values align with the underlying trait they are intended to measure. This means transforming the scores of items like "I don't talk a lot" so that a high score reflects the same trait intensity as a high score on "I talk to a lot of different people at parties".

F Semantic Similarities within DASS Scale

Supplementary Table 3: For each item of the DASS scale (item to consider), the three most semantically similar items are reported.

Item to Consider	1st Most Similar Item	2nd Most Similar Item	3rd Most Similar Item
I found myself getting upset by quite trivial things.	I found myself getting upset rather easily.	I found myself getting agitated.	I found that I was very irritable.
I was aware of dryness of my mouth.	I was aware of the action of my heart in the absence of physical exertion.	I had difficulty in swallowing.	I perspired noticeably in the absence of high temperatures or physical exertion.
I couldn't seem to experience any positive feeling at all.	I couldn't seem to get any enjoyment out of the things I did.	I was unable to become enthusiastic about anything.	I felt that I had nothing to look forward to.
I experienced breathing difficulty.	I experienced trembling.	I was aware of the action of my heart in the absence of physical exertion.	I perspired noticeably in the absence of high temperatures or physical exertion.
I just couldn't seem to get going.	I couldn't seem to get any enjoyment out of the things I did.	I was unable to become enthusiastic about anything.	I found it difficult to work up the initiative to do things.
I tended to over-react to situations.	I found myself getting upset rather easily.	I felt that I was rather touchy.	I found it hard to calm down after something upset me.
I had a feeling of shakiness.	I experienced trembling.	I had a feeling of faintness.	I felt I was close to panic.
I found it difficult to relax.	I found it hard to wind down.	I found it hard to calm down after something upset me.	I found myself getting agitated.
I found myself in situations that made me so anxious I was most relieved when they ended.	I was worried about situations in which I might panic and make a fool of myself.	I was in a state of nervous tension.	I found myself getting agitated.
I felt that I had nothing to look forward to.	I could see nothing in the future to be hopeful about.	I felt that life wasn't worthwhile.	I felt that life was meaningless.
I found myself getting upset rather easily.	I found myself getting upset by quite trivial things.	I found myself getting agitated.	I found that I was very irritable.
I felt that I was using a lot of nervous energy.	I was in a state of nervous tension.	I felt I was close to panic.	I found that I was very irritable.
I felt sad and depressed.	I felt down-hearted and blue.	I felt I was pretty worthless.	I felt that life wasn't worthwhile.
I found myself getting impatient when I was delayed in any way.	I found myself getting agitated.	I found it difficult to tolerate interruptions to what I was doing.	I was intolerant of anything that kept me from getting on with what I was doing.
I had a feeling of faintness.	I had a feeling of shakiness.	I felt I was close to panic.	I felt terrified.
I felt that I had lost interest in just about everything.	I was unable to become enthusiastic about anything.	I felt that I had nothing to look forward to.	I felt that life wasn't worthwhile.
I felt I wasn't worth much as a person.	I felt I was pretty worthless.	I felt that life wasn't worthwhile.	I felt that life was meaningless.
I felt that I was rather touchy.	I found myself getting upset rather easily.	I found that I was very irritable.	I tended to over-react to situations.
I perspired noticeably in the absence of high temperatures or physical exertion.	I experienced trembling.	I experienced breathing difficulty.	I was aware of the action of my heart in the absence of physical exertion.
I felt scared without any good reason.	I felt terrified.	I felt I was close to panic.	I had a feeling of faintness.
I felt that life wasn't worthwhile.	I felt that life was meaningless.	I felt I was pretty worthless.	I felt I wasn't worth much as a person.
I found it hard to wind down.	I found it difficult to relax.	I found it hard to calm down after something upset me.	I found myself getting agitated.
I had difficulty in swallowing.	I experienced breathing difficulty.	I had a feeling of faintness.	I was aware of dryness of my mouth.
I couldn't seem to get any enjoyment out of the things I did.	I was unable to become enthusiastic about anything.	I couldn't seem to experience any positive feeling at all.	I felt that I had lost interest in just about everything.
I was aware of the action of my heart in the absence of physical exertion.	I experienced breathing difficulty.	I perspired noticeably in the absence of high temperatures or physical exertion.	I experienced trembling.
I felt down-hearted and blue.	I felt sad and depressed.	I felt I was pretty worthless.	I felt that life wasn't worthwhile.
I found that I was very irritable.	I found myself getting upset rather easily.	I found myself getting agitated.	I found myself getting upset by quite trivial things.
I felt I was close to panic.	I was in a state of nervous tension.	I felt terrified.	I was worried about situations in which I might panic and make a fool of myself.
I found it hard to calm down after something upset me.	I found myself getting upset rather easily.	I found it hard to wind down.	I found it difficult to relax.

Supplementary Table 3: For each item of the DASS scale (item to consider), the three most semantically similar items are reported.

Item to Consider	1st Most Similar Item	2nd Most Similar Item	3rd Most Similar Item
I feared that I would be thrown by some trivial but unfamiliar task.	I was worried about situations in which I might panic and make a fool of myself.	I felt terrified.	I felt I was close to panic.
I was unable to become enthusiastic about anything.	I felt that I had lost interest in just about everything.	I couldn't seem to get any enjoyment out of the things I did.	I couldn't seem to experience any positive feeling at all.
I found it difficult to tolerate interruptions to what I was doing.	I was intolerant of anything that kept me from getting on with what I was doing.	I found myself getting impatient when I was delayed in any way.	I found myself getting agitated.
I was in a state of nervous tension.	I felt that I was using a lot of nervous energy.	I felt I was close to panic.	I felt terrified.
I felt I was pretty worthless.	I felt I wasn't worth much as a person.	I felt that life wasn't worthwhile.	I felt that life was meaningless.
I was intolerant of anything that kept me from getting on with what I was doing.	I found it difficult to tolerate interruptions to what I was doing.	I found myself getting impatient when I was delayed in any way.	I found that I was very irritable.
I felt terrified.	I felt I was close to panic.	I felt scared without any good reason.	I was in a state of nervous tension.
I could see nothing in the future to be hopeful about.	I felt that I had nothing to look forward to.	I was unable to become enthusiastic about anything.	I felt that life was meaningless.
I felt that life was meaningless.	I felt that life wasn't worthwhile.	I felt I was pretty worthless.	I felt that I had nothing to look forward to.
I found myself getting agitated.	I found myself getting upset rather easily.	I found that I was very irritable.	I found myself getting upset by quite trivial things.
I was worried about situations in which I might panic and make a fool of myself.	I felt I was close to panic.	I feared that I would be thrown by some trivial but unfamiliar task.	I found myself in situations that made me so anxious I was most relieved when they ended.
I experienced trembling.	I had a feeling of shakiness.	I perspired noticeably in the absence of high temperatures or physical exertion.	I experienced breathing difficulty.
I found it difficult to work up the initiative to do things.	I was unable to become enthusiastic about anything.	I just couldn't seem to get going.	I found it difficult to relax.

G PHQ-9 and GAD-7 Items

Supplementary Table 4: PHQ-9 and GAD-7 Assessment Items (rewritten in first person)

Item	Description
	**PHQ-9: Patient Health Questionnaire-9**
PHQ-1	I have little interest or pleasure in doing things
PHQ-2	I feel down, depressed, or hopeless
PHQ-3	I have trouble falling or staying asleep, or I sleep too much
PHQ-4	I feel tired or have little energy
PHQ-5	I have poor appetite or I overeat
PHQ-6	I feel bad about myself — or that I am a failure or have let myself or my family down
PHQ-7	I have trouble concentrating on things, such as reading the newspaper or watching television
PHQ-8	I move or speak so slowly that other people could have noticed, or I am so fidgety or restless that I have been moving a lot more than usual
PHQ-9	I have thoughts that I would be better off dead, or thoughts of hurting myself in some way
	**GAD-7: Generalized Anxiety Disorder-7**
GAD-1	I feel nervous, anxious, or on edge
GAD-2	I am not able to stop or control worrying
GAD-3	I worry too much about different things
GAD-4	I have trouble relaxing
GAD-5	I am so restless that it's hard to sit still
GAD-6	I become easily annoyed or irritable
GAD-7	I feel afraid as if something awful might happen

References

[1] Lovibond, P.F., Lovibond, S.H.: The structure of negative emotional states: Comparison of the depression anxiety stress scales (dass) with the beck depression and anxiety inventories. Behaviour research and therapy **33**(3), 335–343 (1995)

[2] Antony, M.M., Bieling, P.J., Cox, B.J., Enns, M.W., Swinson, R.P.: Psychometric properties of the 42-item and 21-item versions of the depression anxiety stress scales in clinical groups and a community sample. Psychological assessment **10**(2), 176 (1998)

[3] Hendrycks, D., Gimpel, K.: Gaussian error linear units (gelus). arXiv preprint arXiv:1606.08415 (2016)
